# Inadequate soft tissue coverage and bone loss/comminution are the typical risk factors of surgical site infection in open fractures of the hand: A nomogram prediction model

**DOI:** 10.1371/journal.pone.0313832

**Published:** 2025-01-08

**Authors:** Tianyang Jia, Jinglan He, Cunliang Miao, Yubin Long, Qi Dong, Jialiang Guo, Wei Chen, Zhiyong Hou

**Affiliations:** 1 Sixth Department of Orthopedics Surgery, Handan Central Hospital, Handan, Hebei, China; 2 Department of Orthopaedic Surgery, Hebei Medical University Third Hospital, Shijiazhuang, China; 3 Engineering Research Center of Orthopedic Minimally Invasive Intelligent Equipment, Ministry of Education, China; 4 Key Laboratory of Biomechanics of Hebei Province, Shijiazhuang, China; 5 NHC Key Laboratory of Intelligent Orthopeadic Equipment, Shijiazhuang, China; National Trauma Research Institute, AUSTRALIA

## Abstract

**Background:**

Different from other parts of long bone fractures, surgical site infections (SSI) often occurs in open fractures of the hand (OFH) due to the anatomical characteristics and injury mechanisms. Our aim of the study is to investigate the particular risk factors of SSI after emergency surgery in OFH and develop a prediction nomogram model.

**Methods:**

In our traumatic center, patients with OFH not less than 18 years old were retrieved between October 2020 and April 2024. We excluded patients with other fractures, non-traumatic fractures or surgery before admission. The data of these patients were processed by univariate and multivariate analysis using SPSS (24.0) in order to identify the independent risk factors for SSI. Based on the predictors, the nomogram was constructed and validated by R software (R 4.1.0).

**Results:**

The incidence of SSI was 6.96% (43/618). Body mass index (BMI), albumin (ALB), neutrophils (NEU), inadequate soft tissue coverage, and bone loss/comminution were identified as the independent risk factors of post-operative SSI in OFH and enrolled in the prediction nomogram model. The nomogram exhibited a high level of discrimination, with an area under the curve of 0.856 (95%CI 0.790–0.921) in the training group and 0.931 (95%CI 0.848–1.000) in the test group. Hosmer-Lemeshow (H-L) test revealed optimal consistency between the probability of prediction model and the actual probability (training group: X2 = 5.706, P = 0.680; test group: X2 = 3.886, P = 0.867). The calibration curve of both groups demonstrated excellent consistency. Decision curve analysis (DCA) showed favorable applicability of the prediction model.

**Conclusions:**

Inadequate soft tissue coverage, serum ALB level, NEU level, bone loss/comminution and BMI were the independent risk factors for post-operative SSI in OFH. The nomogram of this predictors can be used as an effective tool to predict SSI risk in OFH.

## Introduction

Open fractures of the hand (OFH) are one of the most common orthopedic traumas presenting to emergency departments [1–3]. According to data from a 10-year national investigation, about 2.6 million patients with hand traumatic injuries visited American Emergency Departments every year over the decade 2009–2018 [4]. Compared with closed fractures of the hands, OFH is often associated with soft tissue damage and contamination by foreign bodies or pathogenic bacteria, increasing the risk of surgical site infections (SSI). Moreover, different from other parts of open fractures, inadequate soft tissue coverage, bone loss/comminution, neurovascular injury, tendon injury are more likely to occur in OFH due to the anatomical characteristics and injury mechanisms. A meta-analysis involving 201 studies and 315,618 patients indicated that the incidence of SSI in OFH was between 5% and 10% [5], which might be even higher in the past. Post-operative SSI in OFH can lead to prolonged healing and hospital stays with different degrees of complications, including stiffness, soft tissue defects, osteomyelitis and even amputation, requiring repeated debridement and irrigation due to deep musculoskeletal infection [6]. A study of the European health care systems revealed that the extended length of hospital stay associated with an SSI was about 7 to 14 days and the cost burdens could be up to £19.1 billion a year in Europe [7].

Therefore, a prediction model suitable for OFH is necessary for clinicians to take preventive measures to reduce the probability and cost burden of post-operative SSI and to improve the prognosis after surgery. As a visual prediction tool, a nomogram matches independent risk factors with a weight number and shows complex mathematical equations in a simple chart. At present, the nomogram is widely utilized to predict the diagnosis, prognosis and complications of various diseases [8–10], which has an integrated system for evaluating performance, discrimination, and applicability of the prediction model.

Risk factors of post-operative SSI in OFH have been investigated in several studies [11–16], but none of these studies used a nomogram to predict the incidence of infections. Hence, a retrospective cohort study was worked out for two aims: (1) to investigate and analyze the epidemiological characters and preoperative particular risk factors of SSI following emergency surgery in OFH; (2) to develop and evaluate a predictive nomogram model to help clinicians identify OFH with a high risk of SSI after surgery.

## Methods

### Patients

The study obeyed the ethical principles proposed in the Helsinki Declaration. The Hospital Ethics Committee approved our research (KY2023-0030-01). The informed consent was exempt. The individual information of the patients was deleted, and the data was anonymous. We collected the records of patients in OFH following emergency surgery from October 2020 to April 2024 and analyzed their medical data. Inclusion criteria: (1) the diagnosis was OFH; (2) adults (not less than 18 years old); (3) the complete individual data was available. Exclusion criteria: (1) the diagnosis included other fractures; (2) non-traumatic fractures, such as closed fractures or pathological fractures; (3) received other irrigation, debridement or other surgeries before admission; (4) bilateral fractures; (5) amputation or finger ischemia requiring revascularization; (6) post-operative follow-up was less than 30 days.

### Data collection

The dependent variable was whether or not SSI occurred after emergency surgery. Infected wounds would be cultured for secretion bacteria and then we recorded the pathogenic bacteria. We completed the collection of relevant data in June 2024. We identified SSI after surgery in OFH following the Guidelines for Prevention of SSI 1999 [17]. Variables included 3 categories and 26 items: (1) general data (11 items): age, gender, body mass index (BMI), admission season, pre-admission time, cause of injury (cut, laceration, crush, others), smoking history, drinking history, diabetes, hypertension, American Society of Anesthesiologists (ASA) score; (2) physical examination (5 items): side, inadequate soft tissue coverage, bone loss/comminution, neurovascular injury, tendon injury; (3) laboratory tests (10 items): red blood cell (RBC), white blood cell (WBC), blood platelet (PLT), hemoglobin (HB), neutrophil (NEU), lymphocyte (LYM), total protein (TP), globulin (GLOB), albumin (ALB) and admission blood glucose (GLU). All data of participants was reviewed and collected by two orthopedic surgeons. Another senior surgeon in our group discussed any uncertain data to reach an agreement. At 1, 2 and 4 weeks after discharge, patients were reviewed in the outpatient department or were routinely followed up by phone calls.

### Statistical analysis

The data was analyzed by SPSS (24.0) and R software (R 4.1.0). The mean ± standard deviation (SD) was used to record the continuous variables. Distribution normality was examined by the Shapiro-Wilk test. According to the examination of distribution normality, a Whitney U test or t test was used to analyze the continuous variables. A chi-square test was used to analyze the categorical variables. In the univariate analysis, the variables showing p<0.05 were next admitted to the multivariate stepwise logistic analysis. In the multivariate analysis, the variables showing p<0.05 were identified as the independent risk factors of SSI in OFH after emergency surgery and were used to develop the prediction nomogram chart. The consistency and predictive performance of the model were evaluated by the receiver operating characteristic (ROC) curve, decision curve analysis (DCA) and calibration curve. The Hosmer-Lemeshow (H-L) test was used to evaluate the goodness-of-fit of the nomogram.

## Results

### Baseline characteristics

**Fig 1:**
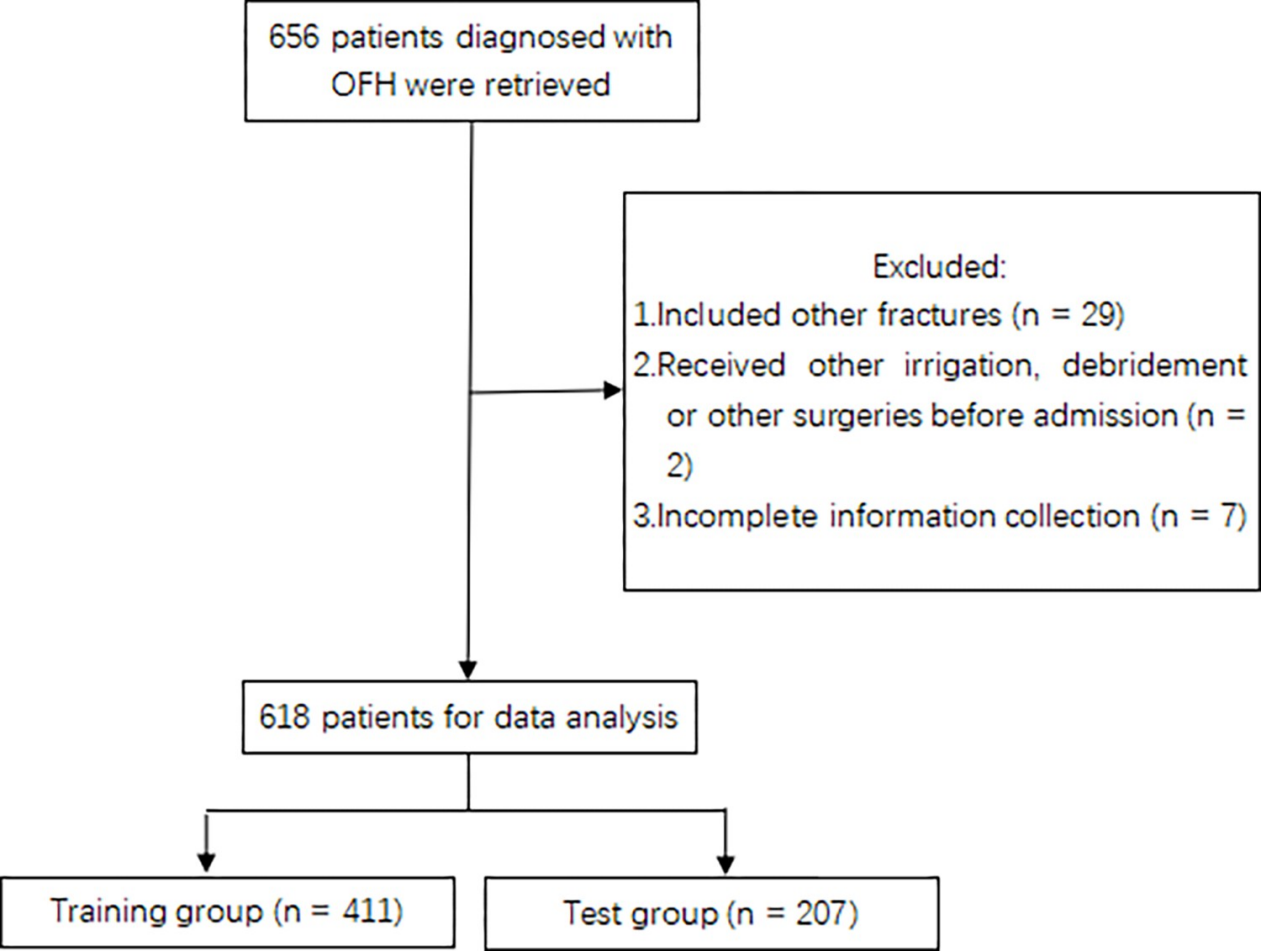
Workflow for the selection of the study participants.

656 patients diagnosed with OFH were retrieved and 38 were excluded for different reasons (Fig 1). All patients with OFH were given antibiotics to prevent infection no later than 30 minutes after operation. The clinical data of 618 patients was randomly assigned to a training group (n = 411) or a test group (n = 207) with a 7:3 ratio by R software. In this study, the SSI incidence of all 618 OFH patients following emergency surgery was 6.96% (43/618). The most common infection strain of pathogenic bacteria was Pseudomonas aeruginosa (9/43, 20.93%). Statistically significant differences were not found between the cohorts for any variables (Table 1, all P > 0.05).

**Table 1:** Comparison of baseline data between training group and test group.

Variable	Training group (n = 420)	Test group (n = 198)	χ^2^/t	P
Age, year (mean [SD])	41.60 (13.50)	43.72 (13.13)	-1.837	0.067
Gender, n (%)			1.135	0.285
Male	314 (74.8)	140 (70.7)		
Female	106 (25.2)	58 (29.3)		
BMI, kg/m2 (mean [SD])	24.76 (3.26)	24.26 (3.36)	1.737	0.083
Admission season, n (%)			1.958	0.581
Spring	98 (23.3)	50 (25.3)		
Summer	114 (27.1)	59 (29.8)		
Fall	115 (27.4)	44 (22.2)		
Winter	93 (22.1)	45 (22.7)		
Pre-admission time, n (%)			0.294	0.636
<6 hours	385 (91.7)	184 (92.9)		
≥ 6 hours	35 (8.3)	14 (7.1)		
Cause of injury			3.117	0.374
Cut	230 (54.8)	105 (53.0)		
Laceration	25 (6.0)	10 (5.1)		
Crush	159 (37.9)	76 (38.4)		
Others	6 (1.4)	7 (3.5)		
Smoking history, n (%)			0.305	0.629
Yes	112 (26.7)	57 (28.8)		
No	308 (73.3)	141 (71.2)		
Drinking history, n (%)			1.128	0.320
Yes	100 (23.8)	55 (27.8)		
No	320 (76.2)	143 (72.2)		
Diabetes, n (%)			2.094	0.164
Yes	40 (9.5)	12 (6.1)		
No	380 (90.5)	186 (93.9)		
Hypertension, n (%)			0.032	0.911
Yes	76 (18.1)	37 (18.7)		
No	344 (81.9)	161 (81.3)		
ASA score, n (%)			0.940	0.362
<3	316 (75.2)	156 (78.8)		
≥ 3	104 (24.8)	42 (21.2)		
Side, n (%)			0.121	0.730
Left	229 (54.5)	105 (53.0)		
Right	191 (45.5)	93 (47.0)		
Inadequate soft tissue coverage, n (%)			0.249	0.733
Yes	30 (7.1)	12 (6.1)		
No	390 (92.9)	186 (93.9)		
Bone loss/comminution, n (%)			0.177	0.667
Yes	40 (9.5)	21 (10.6)		
No	380 (90.5)	177 (89.4)		
Neurovascular injury, n (%)			0.005	1.000
Yes	179 (42.6)	85 (42.9)		
No	241 (57.4)	113 (57.1)		
Tendon injury, n (%)			0.160	0.730
Yes	213 (50.7)	97 (49.0)		
No	207 (49.3)	101 (51.0)		
WBC, n (%)			0.100	0.777
<10×10^9^/L	296 (70.5)	142 (71.7)		
≥ 10×10^9^/L	124 (29.5)	56 (28.3)		
NEU, n (%)			0.466	0.546
<6.3×10^9^/L	216 (51.4)	96 (48.5)		
≥ 6.3×10^9^/L	204 (48.6)	102 (51.5)		
LYM, n (%)			0.722	0.697
<1.8×10^9^/L	301 (71.7)	144 (72.7)		
(1.8–3.2) ×10^9^/L	112 (26.7)	49 (24.7)		
≥ 3.2×10^9^/L	7 (1.7)	5 (2.5)		
RBC, n (%)			0.366	0.635
<lower limit	13 (3.1)	8 (4.0)		
≥ lower limit	407 (96.9)	190 (96.0)		
PLT, n (%)			0.011	0.919
(100–300) ×10^9^/L	324 (77.1)	152 (76.8)		
(<100 or>300) ×10^9^/L	96 (22.9)	46 (23.2)		
HB, n (%)			1.298	0.287
<lower limit	29 (6.9)	9 (4.5)		
≥ lower limit	391 (93.1)	189 (95.5)		
TP, n (%)			0.017	1.000
<55g/L	14 (3.3)	7 (3.5)		
≥ 55 g/L	406 (96.7)	191 (96.5)		
ALB, n (%)			0.945	0.374
<35 g/L	32 (10.0)	15 (7.6)		
≥ 35 g/L	378 (90.0)	183 (92.4)		
GLOB, n (%)			0.982	0.336
<30 g/L	339 (80.7)	153 (77.3)		
≥ 30 g/L	81 (19.3)	45 (22.7)		
admission GLU, n (%)			0.043	0.857
<6.1mmol/L	273 (65.0)	127 (64.1)		
≥ 6.1 mmol/L	147 (35.0)	71 (35.9)		

Abbreviations: BMI, body mass index; ASA, American Society of Anesthesiologists; WBC, white blood cell; NEU, neutrophil; LYM, lymphocyte; RBC, red blood cell, reference range: female, 3.5–5.0×10^12^/L; male, 4.0–5.5×10^12^/L; PLT, blood platelet; HB, hemoglobin, reference range: female, 110-150g/L; male, 120-160g/L; TP, total protein; ALB, albumin; GLOB, globulin; GLU, glucose.

### Univariate analysis of risk factors for SSI in OFH

Table 2 presents the comparison of each variable between the training group and the test group. BMI, bone loss/comminution, inadequate soft tissue coverage, neurovascular injury, WBC level, NEU level, TP level and ALB level had a significant relationship with the incidence of SSI in OFH following emergency surgery (P <0.05).

**Table 2:** Univariate analysis for risk factors of SSI in OFH in training group.

Variables	Non-SSI group (n = 386)	SSI group (n = 34)	χ^2^/t	P
Age, year (mean [SD])	41.08 (13.44)	47.50 (12.90)	-2.677	0.008*
Gender, n (%)			0.030	0.839
Male	289 (74.9)	25 (73.5)		
Female	97 (25.1)	9 (26.5)		
BMI, kg/m2 (mean [SD])	24.64 (3.23)	26.06 (3.42)	-2.441	0.015*
Admission season, n (%)			1.570	0.666
Spring	90 (23.3)	8 (23.5)		
Summer	102 (26.4)	12 (35.3)		
Fall	108 (28.0)	7 (20.6)		
Winter	86 (22.3)	7 (20.6)		
Pre-admission time, n (%)			0.570	0.511
<6 hours	335 (92.0)	30 (88.2)		
≥ 6 hours	31 (8.0)	4 (11.8)		
Cause of injury			1.349	0.691
Cut	212 (54.9)	18 (52.9)		
Laceration	23 (6.0)	2 (5.9)		
Crush	146 (37.8)	13 (38.2)		
Others	5 (1.3)	1 (2.9)		
Smoking history, n (%)			0.143	0.689
Yes	102 (26.4)	10 (29.4)		
No	284 (73.6)	24 (70.6)		
Drinking history, n (%)			0.002	1.000
Yes	92 (23.8)	8 (23.5)		
No	294 (76.2)	26 (76.5)		
Diabetes, n (%)			2.833	0.119
Yes	34 (8.8)	6 (17.6)		
No	352 (91.2)	28 (82.4)		
Hypertension, n (%)			0.155	0.647
Yes	69 (17.9)	7 (20.6)		
No	317 (82.1)	27 (79.4)		
ASA score, n (%)			0.429	0.536
<3	292 (75.6)	10 (29.4)		
≥ 3	94 (24.4)	24 (70.6)		
Side, n (%)			0.037	0.859
Left	211 (54.7)	18 (52.9)		
Right	175 (45.3)	16 (47.1)		
Inadequate soft tissue coverage, n (%)			76.254	<0.001*
Yes	15 (3.9)	15 (44.1)		
No	371 (96.1)	19 (55.9)		
Bone loss/comminution, n (%)			51.380	<0.001*
Yes	25 (6.5)	15 (44.1)		
No	361 (93.5)	19 (55.9)		
Neurovascular injury, n (%)			9.476	0.003*
Yes	156 (40.4)	23 (67.6)		
No	230 (59.6)	11 (32.4)		
Tendon injury, n (%)			2.898	0.108
Yes	191 (49.5)	22 (64.7)		
No	195 (50.5)	12 (35.3)		
WBC, n (%)			9.750	0.003*
<10×10^9^/L	280 (72.5)	16 (47.1)		
≥ 10×10^9^/L	106 (27.5)	18 (52.9)		
NEU, n (%)			16.901	<0.001*
<6.3×10^9^/L	210 (54.4)	6 (17.6)		
≥ 6.3×10^9^/L	176 (45.6)	28 (82.4)		
LYM, n (%)			2.880	0.215
<1.8×10^9^/L	272 (70.5)	29 (85.3)		
(1.8–3.2) ×10^9^/L	107 (27.7)	5 (14.7)		
≥ 3.2×10^9^/L	7 (1.8)	0 (0.0)		
RBC, n (%)			0.958	0.284
<lower limit	11 (2.8)	2 (5.9)		
≥ lower limit	375 (97.2)	32 (94.1)		
PLT, n (%)			0.570	0.529
(100–300) ×10^9^/L	296 (76.7)	28 (82.4)		
(<100 or >300) ×10^9^/L	90 (23.3)	6 (17.6)		
HB, n (%)			1.359	0.278
<lower limit	25 (6.5)	4 (11.8)		
≥ lower limit	361 (93.5)	30 (88.2)		
TP, n (%)			8.162	0.020*
<55g/L	10 (2.6)	4 (11.8)		
≥ 55 g/L	376 (97.4)	30 (88.2)		
ALB, n (%)			26.299	<0.001*
<35 g/L	30 (7.8)	12 (35.3)		
≥ 35 g/L	356 (92.2)	22 (64.7)		
GLOB, n (%)			0.428	0.500
<30 g/L	313 (81.1)	26 (76.5)		
≥ 30 g/L	73 (18.9)	8 (23.5)		
admission GLU, n (%)			1.352	0.263
<6.1mmol/L	254 (65.8)	19 (55.9)		
≥ 6.1 mmol/L	132 (34.2)	15 (44.1)		

*P value<0.05

Abbreviations: BMI, body mass index; ASA, American Society of Anesthesiologists; WBC, white blood cell; NEU, neutrophil; LYM, lymphocyte; RBC, red blood cell, reference range: female, 3.5–5.0×10^12^/L; male, 4.0–5.5×10^12^/L; PLT, blood platelet; HB, hemoglobin, reference range: female, 110-150g/L; male, 120-160g/L; TP, total protein; ALB, albumin; GLOB, globulin; GLU, glucose.

### Multivariate analysis of risk factors for SSI in OFH

Table 3 presents the outcomes of multivariate analysis, which revealed that BMI, ALB, NEU, inadequate soft tissue coverage and bone loss/comminution were independent risk factors and predictors for the incidence of SSI in OFH (P<0.05).

**Table 3:** Multivariate analysis for risk factors of SSI in OFH in training group.

Variables	B	SE	Waldχ^2^	P	OR	95%CI
BMI	0.146	0.067	4.754	0.029*	1.157	1.015–1.320
Bone loss/comminution	1.344	0.535	6.316	0.012*	3.834	1.344–10.938
Inadequate soft tissue coverage	1.704	0.552	9.522	0.002*	5.495	1.862–16.215
NEU≥6.3×10^9^/L	1.407	0.517	7.406	0.007*	4.085	1.482–11.255
ALB<35 g/L	1.477	0.499	8.761	0.003*	4.379	1.647–11.641
Constant	-7.915	1.842	18.465	0.000*		

*P value<0.05

### Nomogram development and validation

**Fig 2:**
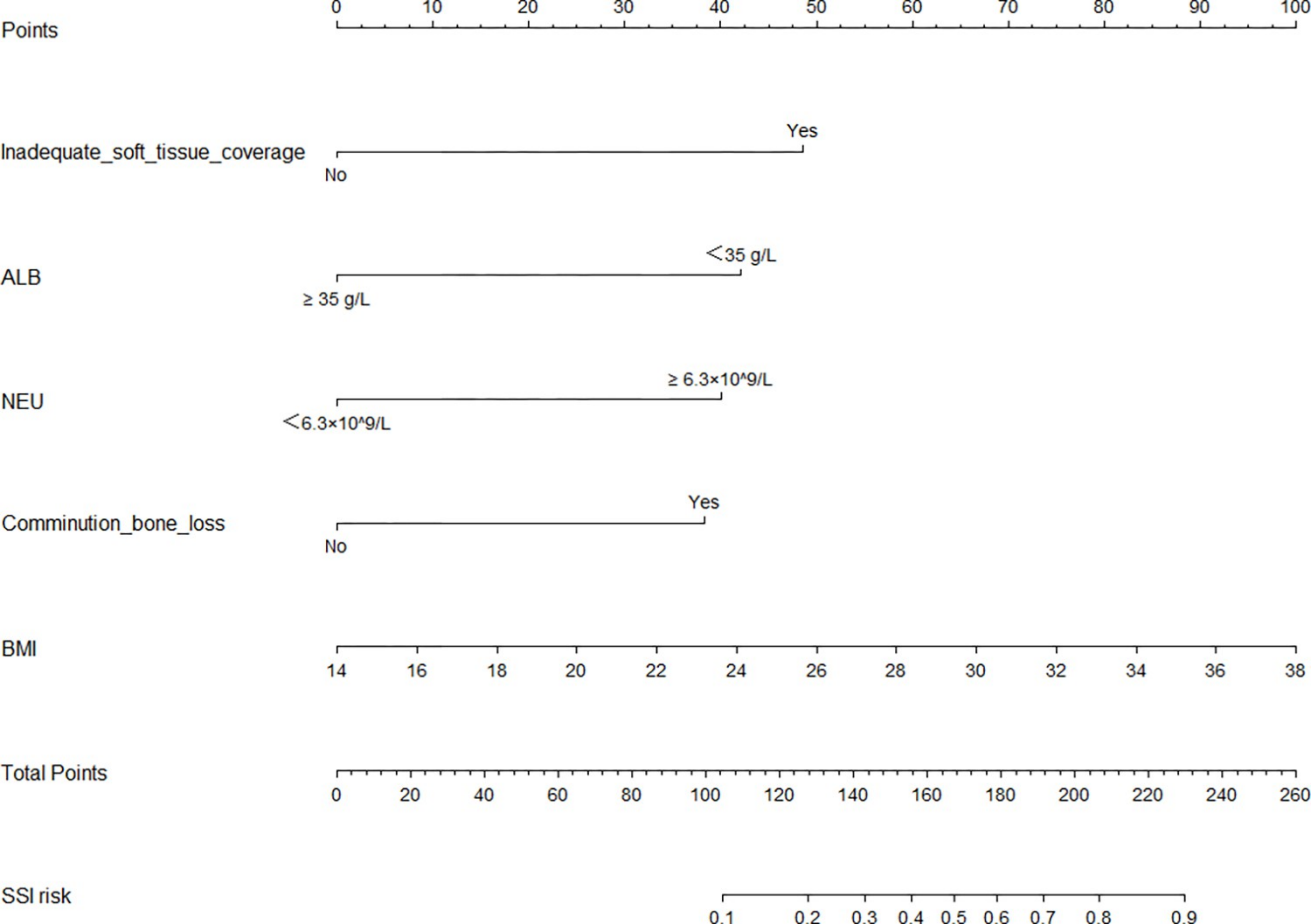
A nomogram prediction model of SSI in OFH.

**Fig 3:**
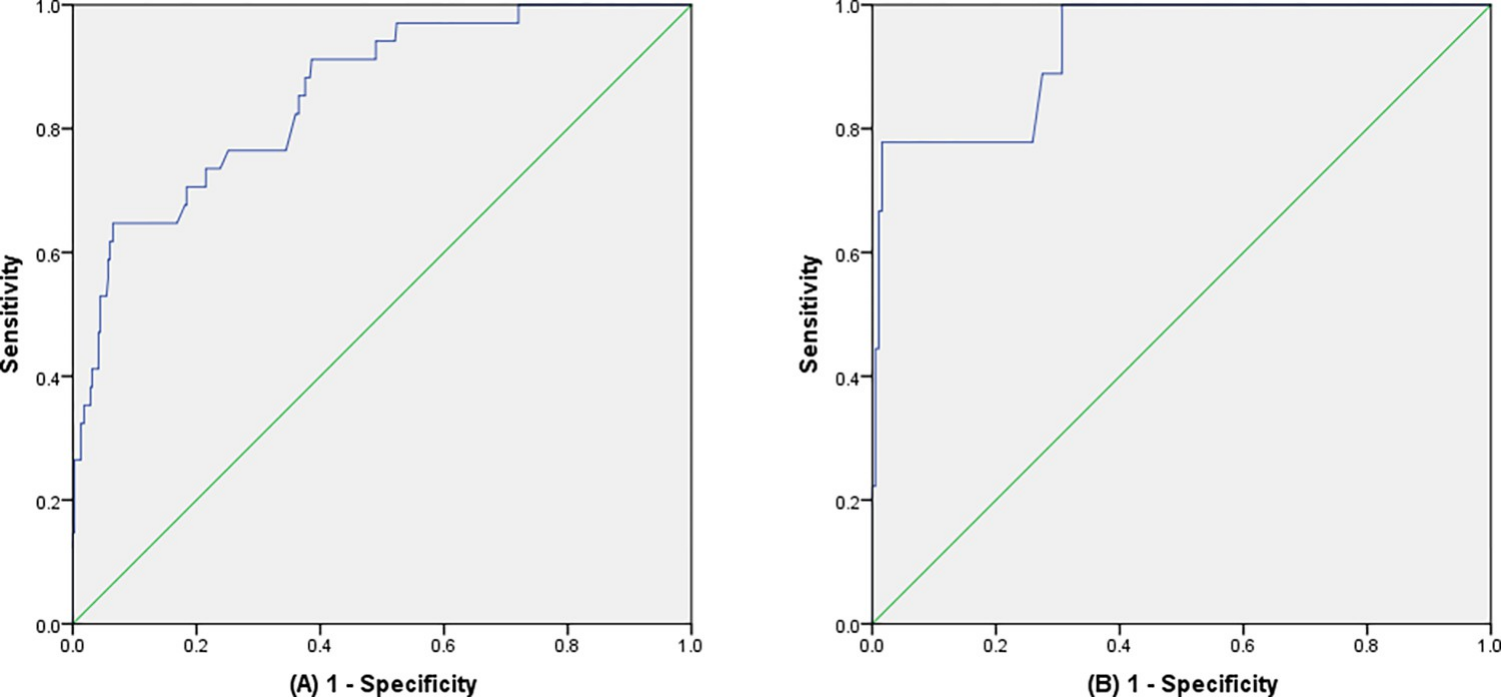
Nomogram prediction model was evaluated by the ROC curve. **A** Training group: the AUC of the nomogram was 0.856. **B** Test group: the AUC of the nomogram was 0.931.

**Fig 4:**
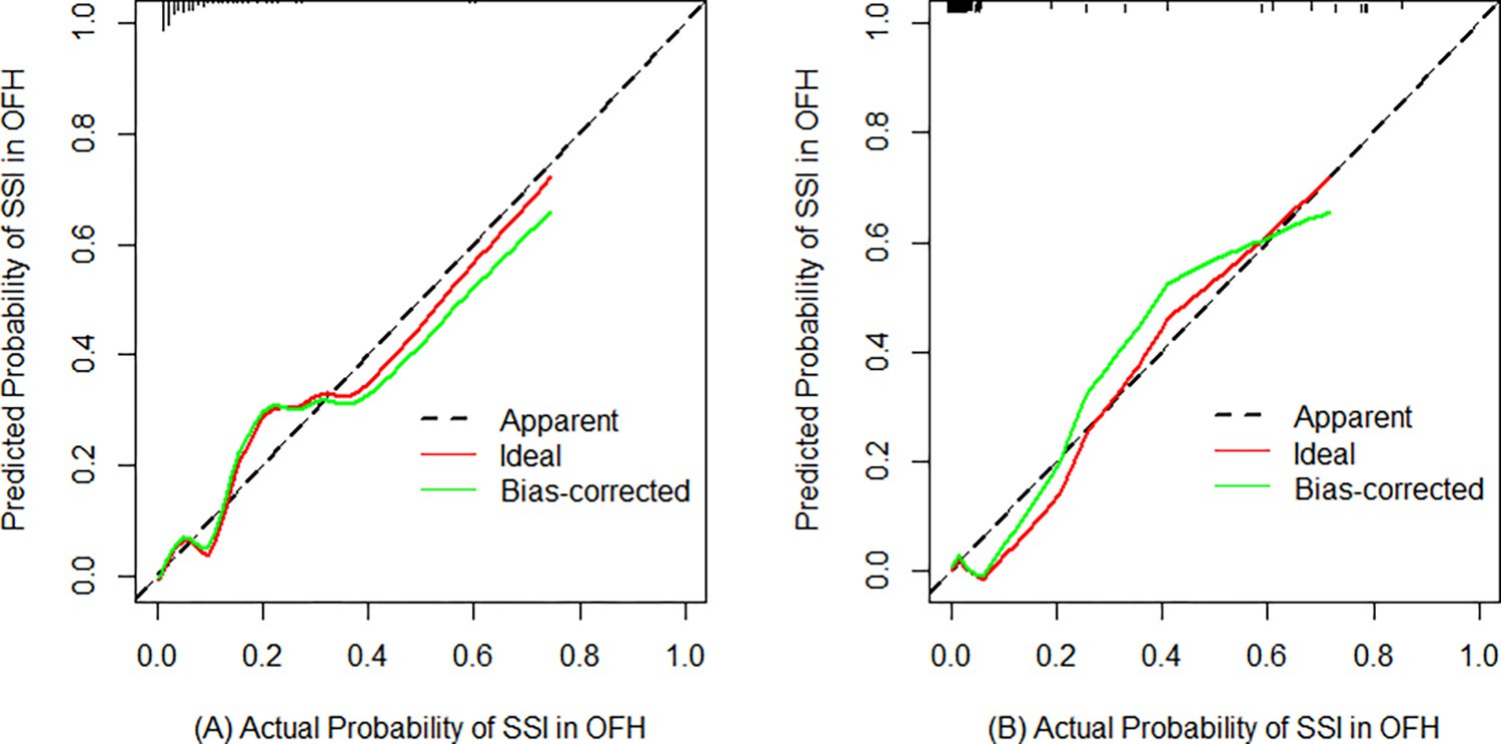
Calibration curve of the nomogram prediction model in the training group. **A** and the test group **B**. SSI: surgical site infection. OFH: open fracture of hands.

**Fig 5:**
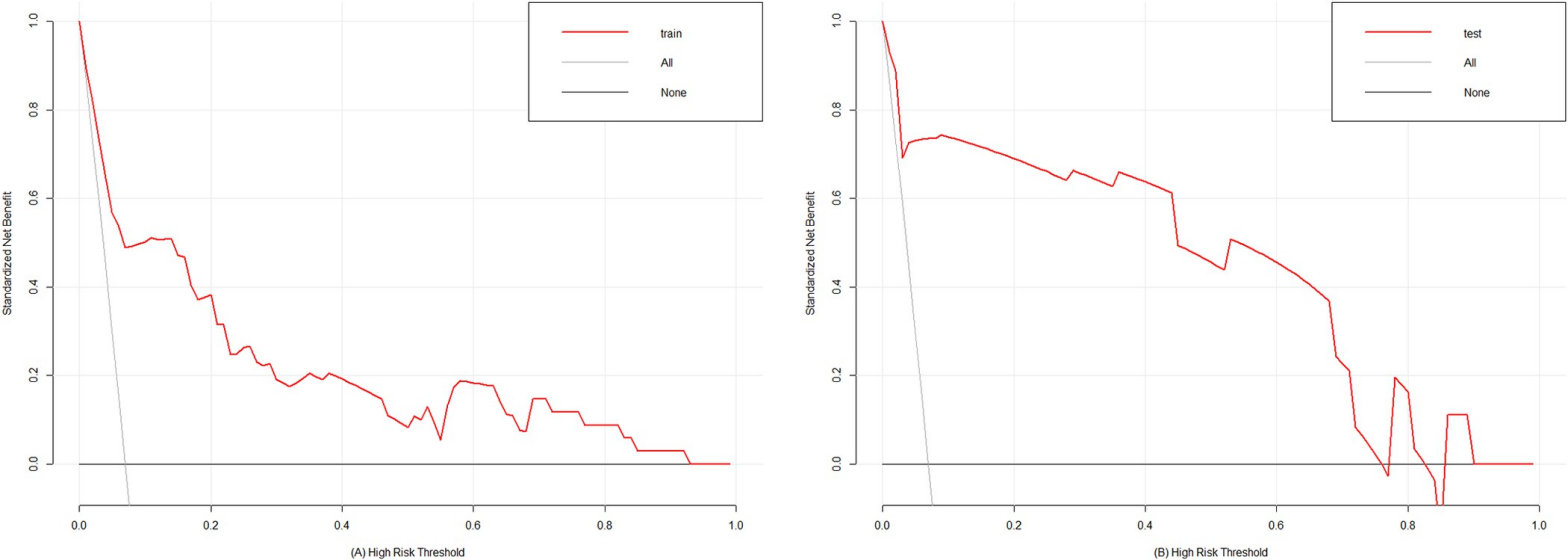
Decision curve analysis (DCA) of the nomogram prediction model in the training group **A** and the test group **B**.

According to the results of the univariate analysis and the multivariate regression analysis, we finally developed a nomogram using the five predictors (Fig 2) and we validated the performance and consistency of the prediction model. The performance of the nomogram was demonstrated by ROC curves (Fig 3). In the training cohort, the area under the curve value (AUC) was 0.856 (95%CI 0.790–0.921), the sensitivity was 0.647, the specificity was 0.935 and the Youden index was 0.582, demonstrating that this model exhibited high predictive discrimination and accuracy. In the test cohort, the AUC was 0.931 (95%CI 0.848–1.000), the sensitivity was 0.778, the specificity was 0.984 and the Youden index was 0.762. The H-L test showed goodness-of-fit (training group: X^2^ = 5.706, P = 0.680; test group: X^2^ = 3.886, P = 0.867). In addition, the calibration curves of two groups indicated optimal consistencies between clinical SSI incidence and predictive probability (Fig 4). Besides, the nomogram DCA curves of two groups showed that the prediction model had excellent applicability for surgeons to predict the incidence of SSI in OFH (Fig 5).

### How to use a nomogram

This gram is a tool to predict the incidence of SSI for OFH patients before emergency surgery. On the left side of this nomogram are five predictor factors, such as inadequate soft tissue coverage, ALB, NEU, comminution/ bone loss and BMI. Each variable is assigned a score on a point-scale axis according to the situation of the OFH patient. Total points are obtained by adding these five individual scores. The SSI possibility could be estimated by drawing vertical lines downward to a lower line segment of SSI risk.

## Discussion

In our retrospective cohort study, the incidence of SSI in OFH was 6.96%. It was consistent with the findings of a meta-analysis involving 201 studies and 315618 patients which concluded that the “true” incidence of SSI in OFH was about 5% to 10% [5]. According to the report of Huang Fang, the post-operative infection incidence of open hand injuries was 5.64% and the pathogenic bacteria were mainly Pseudomonas aeruginosa (17.44%), Staphylococcus aureus (15.12%), Escherichia coli (13.95%) [16]. It was similar to the result of our study that Pseudomonas aeruginosa was the most common infection strain (9/43, 20.93%). In reality, however, it is usually different from studies on the pathogenic bacteria causing SSI. Baisheng Wang et al. collected 566 pathogenic bacteria in fracture-related SSI and found that 53% of infection strains were gram-positive bacteria, of which Staphylococcus aureus was the most common [18]. Then, we identified five independent risk factors for SSI of OFH from 26 preoperative variables: inadequate soft tissue coverage, serum ALB level, NEU level, bone loss/comminution and BMI.

According to the anatomical characteristics, injury mechanisms and clinical experience of open hand fractures, we included four physical examination variables in the multivariate analysis: inadequate soft tissue coverage, bone loss/comminution, neurovascular injury and tendon injury. We found that 3 of these 4 factors mentioned above were associated with SSI (p < 0.05), except for the tendon injury (P = 0.108). Inadequate soft tissue coverage and bone loss/comminution were identified as the independent risk factors through multivariate logistic regression analysis. With the consideration of risk factors for infection and timing of debridement, Tulipan and Ilyas proposed a new classification of open hand fractures using fracture location (phalanges, metacarpals or carpus), contamination, inadequate soft tissue coverage and vascular injury as observational variables [15]. Tulipan suggested that the traditional Gustilo-Anderson classification [19, 20], particularly the size of wounds and soft tissue coverage, was not optimal for hand fractures to predict and prevent SSI due to different nuances in the characteristics of the bones, soft tissue coverage and blood supply. Pichitchai Atthakomol et al. agreed with the limitations of the Gustilo-Anderson classification applied to open hand fractures [11]. However, Pichitchai Atthakomol considered that this classification system lacked objective cohort analysis for validation and that the extent of contamination was subjective, while fracture position was difficult to interpret. Dr. Pichitchai also proposed his classification in which the independent risk factors for infection in OFH requiring re-debridement included diabetes or immunocompromised disorders, bite injury, bone loss/comminution, inadequate soft tissue coverage and neurovascular injury. However, this study did not include laboratory tests in the analysis model and ultimately obtained 8 p-values in the univariate analysis.

Serum ALB and TP in the biochemical laboratory examination is one of the routine emergency test items for orthopedic trauma patients at admission. In the result of our study, both ALB and TP are correlated with SSI in univariate analysis; moreover, ALB is ultimately identified as a predictor for SSI in multivariate analysis, which has been confirmed in other research of orthopedic and subspecialty surgery [21–24]. In a recent study about upper extremity fracture, Dong Xin found that patients with serum ALB<40.8 g/L had a 3.6-fold higher rate of SSI than others [25]. Patients with SSI at risk of malnutrition increased the mortality rate by 6.2 fold according to a 3-year follow-up study [26], emphasizing the seriousness of malnutrition in patients with SSI. On the other hand, a study about acute forearm compartment syndrome found that high levels of ALB was a protective factor for SSI after fasciotomy [27]. ALB is a visual and straightforward indicator to evaluate the status of hypoalbuminemia and nutrition. Trauma patients with open fractures are more susceptible to hypoproteinemia and malnutrition due to blood loss. The decrease in colloid osmotic pressure in patients with hypoalbuminemia may cause wound edema and tissue fluid exudation. With increased wound exudation, the injured tissue can be easily invaded by pathogenic bacteria [28]. Another possible reason is that malnutrition leads to a limitation of fibroblast proliferation and collagen synthesis, which ultimately affects wound healing and prolongs the duration of the open wound [29]. Therefore, clinicians should pay more attention to the nutritional status of OFH patients during the perioperative period in order to decrease the risk of SSI.

The close association between BMI-defined obesity and SSI has gradually become a general consensus among clinicians [30–32], which is consistent with the conclusions of our finding. Lei Xie et al. found that high preoperative BMI was one of the predictors for SSI in closed pilon fractures after traditional surgery [33]. The conclusion that BMI can be used to predict post-operative SSI had been proved to be equally applicable in spinal surgery and gastric cancer surgery [34, 35]. However, the exact biological mechanisms that a high BMI increases the risk of SSI are not clearly revealed. An elevated BMI indicates a higher body fat content and an imbalance in metabolism. Excessive adipose tissue is the source of inflammatory cytokines which in turn cause insulin resistance [36]. This state of high blood glucose and chronic inflammation increases the adhesion of bacteria to tissue, especially in wounds, leading to an increased probability of SSI. On the other hand, inflammatory status caused by SSI would impair the nutritional status of the body through a reduction in food intake and microelement absorption. And then a vicious circle formed.

As a convenient, rapidly obtained, inexpensive, nonspecific biomarker of inflammation, NEU has been widely used in the evaluation and prediction of cardiovascular diseases [37], cancer [38], immune diseases [39] and trauma [40]. A study about colon cancer patients undergoing curative laparoscopic surgery showed that NEU levels on both pre-operative and post-operative day 3 were valuable indicators of SSI, including those with anastomotic leakage [41]. NEU is an important component of the innate immune system, which can engulf and lyse microbial cells that have invaded the body through the recruitment and activation of NEU using lysosomal enzymes [42]. However, excessive recruitment to the wound and activation of NEU lead to disruption of normal cells [43]. A study of patients in six hospitals nationwide in Japan showed that the removal of activated leukocytes from the blood circulation could reduce the incidence of SSI following surgery for ulcerative colitis and demonstrated that excessive activation of NEU is associated with SSI [44]. Some other studies have explored the relationship between neutrophils/lymphocyte rate (NLR) and SSI. In a systematic review, Petr Domecky indicated that NEU levels increased via cytokines after surgery trauma while lymphocyte levels decreased [45]. In the study of Liu Dawei, NLR levels instead of NEU levels was identified as a predictor of SSI in ankle fractures [46]. In this study, both NEU levels and LYM levels were included in the statistical analysis. There was no statistically significant difference in LYM between the SSI and non-SSI groups in the univariate analysis (p = 0.40). But the percentage of LYM<1.8 was higher in the SSI group (81.4%, 35/43) than it was in the non-SSI group (71.3%, 410/575). Hypersensitive C-reactive protein is another immune/inflammatory biomarker which is sensitive to the degree of tissue damage and inflammatory state and is not susceptible to gender, age, HB levels or temperature [47]. However, C-reactive protein is not usually included in the preoperative laboratory tests for emergency trauma patients in part of hospitals.

As far as we know, this is the first nomogram in consideration of physical examination factors including inadequate soft tissue coverage, bone loss/comminution, neurovascular injury and tendon injury to predict the incidence of SSI in OFH. All of the five predictors identified above can be rapidly obtained from clinical physical examination and routine laboratory tests within hours after admission even before emergency surgery, so that the predicted infection probability of OFH is timely available by entering the data into a nomogram and is helpful for surgeons to take preoperative or intraoperative measures to reduce the risk of infection. However, the limitations of the study should be discussed. First, several intraoperative variables which affect the incidence of SSI were not included, such as surgical duration, intraoperative blood loss, anesthesia type, tourniquet use, internal fixation type and transfusion. Second, this is a retrospective study and all data was collected from cases. Hence, information bias was inevitable. Third, our study included Chinese patients, which means that the results are only for reference in other countries. Fourth, our development and validation were based on a single traumatic center. Therefore, the prediction model for SSI risk of OFH needs further clinical data in a prospective study with a large multicenter sample to confirm its applicability and reliability.

## Conclusion

The incidence of SSI in OFH following emergency surgery was 6.96% in our study. We identified inadequate soft tissue coverage, bone loss/comminution, serum ALB level, NEU level and BMI as the independent risk factors for post-operative SSI in OFH and translated the combination of these five predictors into a brief and practical nomogram, which is an effective tool for clinicians to predict and prevent the occurrence of SSI in OFH following emergency surgery.

## Supporting information

S1 Data All data used for the analysis in this article. (XLSX)

## Abbreviations

SSI: Surgical site infections
OFH: Open fractures of the hand
ROC: Receiver operating characteristic
DCA: Decision curve analysis
ALB: Albumin
NEU: Neutrophil
BMI: Body mass index
EDs: Emergency departments
ASA: American Society of Anesthesiologists
WBC: White blood cell
LYM: Lymphocyte
RBC: Red blood cell
PLT: Plood platelet
HB: Hemoglobin
TP: Total protein
GLOB: Globulin
GLU: Admission blood glucose
SD: Standard deviation
NLR: Neutrophil/lymphocyte rate
